# Immobilization of Antimicrobial Silver and Antioxidant Flavonoid as a Coating for Wound Dressing Materials

**DOI:** 10.2147/IJN.S230214

**Published:** 2019-12-17

**Authors:** Hien A Tran, Khanh L Ly, Kate E Fox, Phong A Tran, Thi-Hiep Nguyen

**Affiliations:** 1 Queensland University of Technology (QUT), Brisbane, Queensland 4001, Australia; 2 Interface Science and Materials Engineering Group, School of Chemistry, Physics and Mechanical Engineering, QUT, Brisbane, Queensland, Australia; 3 Centre in Regenerative Medicine, QUT, Brisbane, Queensland, Australia; 4 Department of Biomedical Engineering, International University, Vietnam National University- Ho Chi Minh City (VNU-HCM), Ho Chi Minh City 70000, Vietnam; 5 School of Engineering, Centre for Additive Manufacturing, RMIT University, Melbourne, VIC 3001, Australia

**Keywords:** ﻿quercetin, Ag, antioxidant, antibacterial, wound healing

## Abstract

**Purpose:**

The aim of this study is to develop a new coating for wound dressings that is comprised of antimicrobial silver (Ag) and antioxidant flavonoid quercetin (Q).

**Methods:**

Dip-coating was used to apply the coating on cotton gauge as a model dressing. Ag was immobilised using polydopamine as a priming and catalytic layer followed by coating of quercetin that was incorporated in a functionalized polydimethylsiloxane. The coating was investigated using scanning electron microscopy (SEM), energy-dispersive X-ray spectroscopy (EDX) and release assay. The antimicrobial activity of quercetin and Ag was tested against *Staphylococcus aureus* (*S. aureus*). A surgical wound model on mice was used to evaluate the effects of the coated dressing on wound healing rates and tissue histology.

**Results:**

Ag and quercetin showed enhanced antimicrobial activity against *S. aureus* when used in combination. Ag and quercetin were successfully immobilized onto the fibre of the dressing using the dip-coating process. The coating released Ag and quercetin over 8 days and showed strong antioxidant activity. In the wound healing model, complete wound closure was achieved in 12 days in the group receiving coated dressing and was associated with an enhancement in tissue remodelling and neo-angiogenesis and the reduction in tissue inflammation.

**Conclusion:**

These new antimicrobial-antioxidant coatings may be promising in the development of advanced wound care therapies.

## Introduction

Bacterial infection is among the most common and severe complications in the management of wound healing. The use of antimicrobial wound dressings in infection control has emerged as a promising strategy. Several antimicrobial products are commercially available such as Smith and Nephew’s silver (AG)-impregnated dressing (“Acticoat”). The regulation of oxidative stress is another important factor in successful wound healing; however, it has often been overlooked in the development of wound dressings. The inflammation phase plays an important role in wound healing, dictating the rate and the quality of tissue repair. During this period, an elevated level of reactive oxygen species (ROS) from inflammation may, however, lead to excessive oxidative stress, induce apoptosis, and impair the healing process.^1^–^3^

A number of studies have examined the use of antimicrobial and antioxidant coatings in wound dressings; however, only a few studies have sought to develop coatings that have both properties.^4^ This study thus aims to develop a dual antimicrobial-antioxidant coating for wound dressings. Ag was chosen as the antimicrobial agent because it is among the most commonly studied non-drug antimicrobials with a broad bactericidal spectrum.^5^ Ag is highly effective in suspension form and also when immobilised on surfaces.^6^–^9^ Ag ions and nanoparticles were shown to penetrate the bacterial membrane, attack the respiratory pathway and ultimately cause cell death.^5^ Quercetin was chosen as the antioxidant agent because it is one of the most abundant flavonoids with strong antioxidant and anti-inflammatory properties.^10^ The antioxidant activity of quercetin has been attributed to the scavenging of ROS via hydroxyl groups.^11^ Quercetin has also been shown to facilitate the recruitment of leukocytes to the endothelial walls and alleviate damage to tissue by lessening the degranulation of neutrophils, leading to the release of oxidants and inflammatory mediators to the wound site.^12^,^13^

Several methods have commonly been used to apply coatings to dressings such as sputtering coating and physical/chemical vapour deposition; however, these methods generally require complex instrumentation and sometimes are not compatible with dressing materials. In the current study, we aim to develop a versatile dip-coating method for immobilizing Ag and quercetin as coatings on dressing materials. Specifically, cotton gauze (CG) was chosen as the model dressing material. In the first step, we used the PDA-assisted reduction and immobilisation chemistry to immobilize Ag from silver nitrate solution.^8^,^14^,^15^ A number of studies have shown that dopamine auto-oxidation (at an alkaline pH above 8) can coat a layer of nanometre thick (~less than 5 nm) PDA on substrate chemistries such as metals, ceramics, and polymers.^16^–^18^ The newly formed PDA layers were also shown to have the capability to reduce metal ions from a solution in contact into metal submicro-particles/nanoparticles that are immobilised on the PDA surface.^18^ In the next step, quercetin was immobilised by dipping the Ag-coated samples in a solution containing quercetin and a commercial medical grade methoxyl-, amine-functionalised silicone which formed a polymerized coating upon reacting with moisture in the air during drying. This commercially available medical grade silicone dispersion is used clinically to coat cutting edges to reduce friction and has been proven to be a versatile and effective binding agent in immobilising a number of agents onto surfaces.^19^–^21^ This binding agent was also chosen to add lubrication to the dressing, which should prevent the dressing from adhering too strongly to the wound for easy dressing removal or exchange.^22^,^23^

## Materials and Methods

### Materials

NaHCO_3_, Na_2_CO_3_, AgNO_3_, dopamine hydrochloride (DA), quercetin, 2,2-diphenyl-1-picrylhydrazyl (DPPH), isopropanol, methoxyl-, amine-functionalised silicone, haematoxylin and eosin (H&E), and Masson’s trichrome (MT) staining kit were purchased from Sigma-Aldrich, USA. The optimal cutting temperature (OCT) compound known as the inert support medium for cryo-sectioning was obtained from ThermoFisher Scientific, USA. The Zoletil50^®^ was supplied by Virbac, Vietnam. Ferric chloride (FeCl_3_), ethanol, acid acetic and formal aldehyde were purchased from Xilong Chemical Ltd, China. The mice used in the in vivo studies were supplied by the Pasteur Institute, Ho Chi Minh City, Vietnam.

### Preparation of Cotton Gauge (CG) Coated with Quercetin and Ag (CG-Ag-Q)

The coating of the PDA and the deposition of the Ag were modified from process used in previous studies.^14^,^18^,^24^ Briefly, in step 1 (Ag coating), the CG samples (10 mm in length and 10 mm in width) were pre-wetted with a 0.1 M carbonate buffer (pH = 8.5) in a petri dish and covered completely before being placed under vacuum to remove trapped air bubbles. The same volume of DA (1 g/L) solution (0.4 mL of DA stock (100 g/L) diluted in 40 mL carbonate buffer) was added to the petri dishes containing the CG samples. The petri dish was placed on the shaking plate for 60 mins to enable the DA to oxidize and polymerise to form polydopamine (PDA). Notably, the samples turned from a white to a dark brown colour after the process. Next, the samples were transferred to immerse in 10 mM AgNO_3_ for 30 mins. The samples were rinsed four times with distilled water and then dehydrated by immersing in a series of isopropanol dilutions (80%, 90%, and 100%, respectively). Step 2 (quercetin coating), quercetin was dissolved in isopropanol to make 14.4 mg/mL stock solution and an aliquot of 0.45 mL quercetin stock solution was mixed with a solution containing 0.211 mL polydimethylsiloxane and 0.95 mL of 70% hexane/30% isopropanol to make quercetin coating solution. The samples at the end of Step 1 were then dip-coated in the quercetin coating solution and dry overnight at room temperature. The CG and CG-Ag-Q samples were sterilised using ethylene oxide before the in vivo study.

### Material Characterisation

#### Surface Morphology

The surface morphology and chemical composition of the CG, CG-Ag and CG-Ag-Q dressings were studied using scanning electron microscopy (SEM) (JSM-7001F, Jeol). The presence of Ag particles was confirmed using SEM in secondary electron (SE), backscatter electron (BSE) modes and energy dispersive X-ray spectroscopy (EDX). Samples were gold coated (Leica EM SCD005, Leica) before imaging.

#### Release of Ag and Quercetin

The release of Ag and quercetin was studied by incubating CG-Ag-Q sample in 0.5 mL phosphate buffer saline (PBS, pH = 7.4) tubes at 37^o^C. The supernatant was collected daily and the tubes were refilled with fresh PBS. One hundred microliters of supernatant were transferred to a 96-well plate to measure the absorbance at 410 nm using a spectrophotometer (xMark, Biorad) for quercetin concentration quantification. An aliquot of 0.3 mL of supernatant was completely dissolved with 0.3 mL of concentrated nitric acid followed by adding distilled water to the solution to make up the final volume of 10 mL. The solution was analysed using an inductively coupled plasma optical emission spectrometry (ICP-OES, Thermo Fisher Scientific, USA) to determine Ag concentration.

### In vitro Studies

#### Antibacterial Study

A microplate broth culture assay was performed to evaluate the antimicrobial efficacy of the quercetin and Ag-Q in combination against *S. aureus*.^25^ Briefly, the culture purity was confirmed from the subculture plate using Gram stain prior to the experiment. Next, an inoculating loop was used to select several colonies from the subculture plate and transfer them to sterile tube containing 5 mL sterile saline. A bacterial suspension with an optical density at 625 nm (OD_625_) value of 0.1 (equivalent to 0.5 McFarland Standards) was obtained by the dilution. This bacteria suspension was further diluted 100 times in Mueller Hinton (MH) broth and used for inoculation. Fifty microliters of serial dilutions of quercetin only in MH broth were prepared in a sterile 96-well plate to which 50 µL of inoculation solution was added. Another set of serial dilutions of quercetin was prepared and added with Ag to final concentration of 1 µg/mL Ag to test the antimicrobial activity of Ag-Q when used in combination. After 12 hrs of aerobic incubation at 37^o^C, the bacteria inhibition was evaluated by measuring the OD_625_ of the bacteria suspension in a 96-well plate. The experiment was performed in duplicates.

#### Antioxidant Study

The antioxidant activities of the CG-Ag-Q samples were evaluated in relation to the free radical scavenging effect of a stable DPPH based on established protocols.^26^ The assay is based on reduction in DPPH absorbance at a 517-nm wavelength when treated with an antioxidant. Briefly, 4 mg of DPPH was dissolved in ethanol to obtain 0.1 mM DPPH solution. Three samples were weighted and placed in separate vials. To determine the radical scavenging activity, 1 mL DPPH solution was added and mixed in each vial and the vials were shaken and incubated at room temperature. The absorbance of solution at 517nm was measured at 5, 10, 15, 30, 45, 60, 90, and 120 mins using Multi-plate reader (Varioskan^TM^ LUX microplate reader). The experiment was performed in triplicates. The radical scavenging activity of each sample was calculated as the reduction (%) in absorbance compared to the untreated DPPH control.

### In vivo Study

The ethic for this study was approved by the Institutional Animal Care and Use Committee of International University, Vietnam National University, Ho Chi Minh City, Vietnam. The experiments were conducted in compliance with the NIH Guide for Care and Use of Laboratory Animals.

Twelve mice were divided equally into two groups. The mice were administered anaesthesia (Zoletil50^®^ at 20–40 mg/kg) via an intramuscular injection. Next, a round incision (8 mm in diameter) was created on the dorsal skin of the mice. The wounds were covered with dressing samples (1 x 1 cm) and secured firmly using sutures. The wounds in control group were covered with CG samples, and in the treatment group, they were covered with CG-Ag-Q samples. Postsurgery, wound dimensions were recorded at days 7, 10, and 12 and wound closure (%) was calculated as the percentage of wound area that has closed.

At days 7, 10, and 12, two mice from each group were sacrificed and wound tissues were collected. The specimens were embedded in a plastic holder containing OCT compound and stored at –80^o^C prior to cryo-sectioning. Sections (5 µm thick) from each frozen specimens were obtained using cryostat (Thermo Fisher Scientific Inc., USA) and stained using H&E and MT kit. The slides were then imaged using light microscopy (Nikon Eclipse, Ti-U series, Japan).

## Results

### Material Characterization

**Figure 1:**
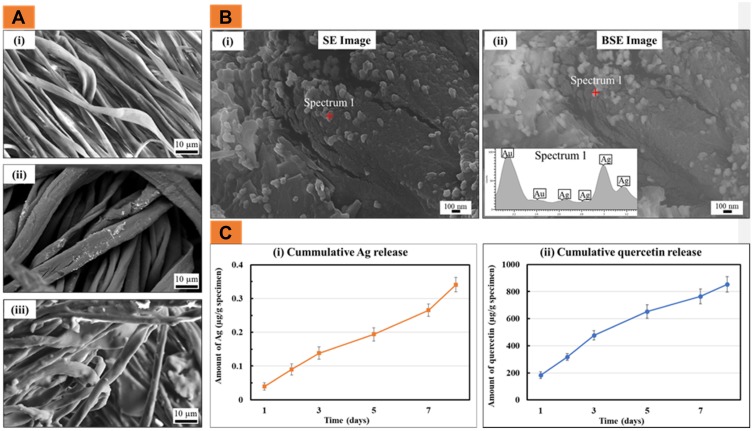
(**A**) The SEM images of (i) CG, (ii) CG-Ag and (iii) CG-Ag-Q; (**B**) The secondary electron (SE) image (i) coupled with the BSE image (ii) and the EDX spot analysis of the CG-Ag samples; (**C**) The release profile of (i) Ag and (ii) quercetin from the CG-Ag-Q (data = mean ± standard error of means; n = 6).

The SEM was used to characterize the changes in morphology after each coating step. Figure 1(A)(i) shows the original CG specimen morphology in which the CG fibres had a smooth surface and were approximately 2–4 µm in diameter. Figure 1(A)(ii) shows the changes in morphology of the CG-Ag as compared to the CG. Specifically, after the polymerisation of the PDA layer, the diameter of the CG fibres increased noticeably to 5–10 µm. EDX analysis and BSE imaging were used to confirm the presence of Ag submicro-/nano-particles on the dressing. Figure 1(B)(i-ii) shows Ag appeared as clusters of roughly 100 nm (SE and BSE images) confirmed by EDX analysis that showed strong Ag peaks (inset b-ii). Finally, Figure 1(A)(iii) shows the smooth morphology of the quercetin-polydimethylsiloxane coating which appeared to cover the Ag coating.

To determine the in vitro release of Ag and quercetin from the CG-Ag-Q samples, the samples were incubated in PBS (pH = 7.4) for 8 days. Figure 1(C)(i) showed that Ag release increased sharply in the first 3 days from 0.05 to 0.15 µg/g of specimen. The release then increased gradually over the next 5 days to approximately 0.275 µg/g. On the eighth day, the release of Ag increased significantly to 0.35 µg/g. Figure 1(C)(ii) shows that on the first day, 200 µg of quercetin was released per 1 g of specimen. On the third day, this amount increased significantly to approximately 500 µg. The quercetin was then released gradually and reached a total of approximately 850 µg per 1 g of the specimen.

### In vitro Studies

**Figure 2:**
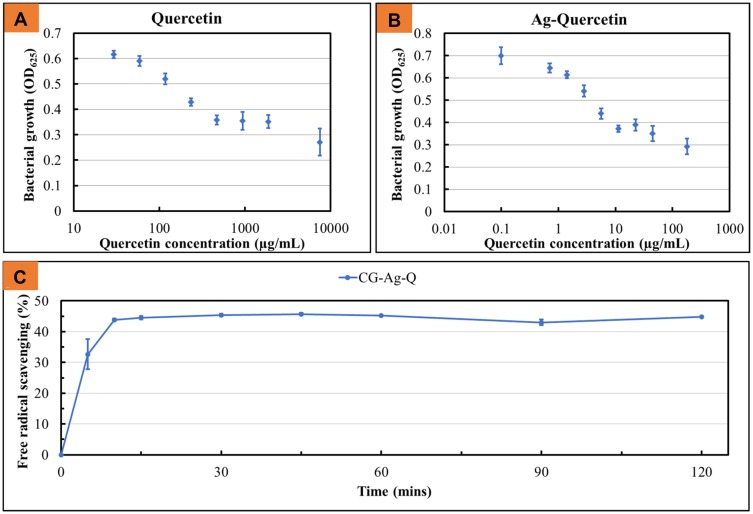
(**A**) Dose-dependent antimicrobial activity of quercetin against *S. aureus*; (**B**) Antimicrobial activity of Ag and quercetin when used in combination (data = mean ± standard error of means; n = 3); and (**C**) Antioxidant activity of the CG-Ag-Q (data = mean ± standard error of means; n = 3).

The antibacterial activity of the quercetin either alone or in combination with Ag against the *S. Aureus* was studied. The results are shown in Figure 2(A, B).

When quercetin was used in combination with Ag at Ag concentration of 1 µg/mL, the bacterial growth was significantly inhibited (as indicated by a lower OD). Specifically, when treated with 10 µg/mL quercetin-only, the OD of the bacteria suspension was 0.6; when treated with Ag-Q at the same quercetin concentration, the OD of the bacteria was approximately 0.4; when treated with quercetin only at 100 µg/mL, the OD of the bacteria was 0.5; and when treated with Ag-Q at the same quercetin concentration, the OD of the bacteria was approximately 0.3. Further, as Figure 2(C) shows, the CG-Ag-Q samples scavenged almost 45% of the free radicals within approximately 10 mins.

### In vivo Study

**Figure 3:**
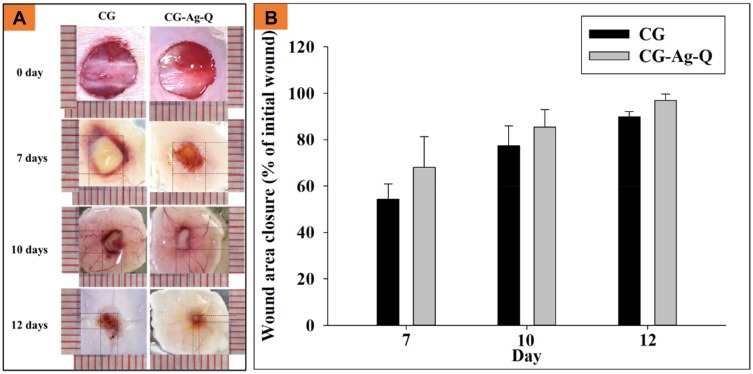
CG-Ag-Q dressing accelerated wound closure. (**A**) Photographs showing wound sites at 0 and 7, 10 and 12 days post-surgery; (**B**) Wound closure (indicated by wound area as % of area at day 0) at 7, 10 and 12 days post-surgery (data = mean ± standard error of means, n=2 biological repeats).

The wound healing performance of the CG-control and CG-Ag-Q group was first evaluated by measuring wound areas and calculating wound closure. Figure 3 shows the photographs (see Figure 3(A)) of the wounds and the graph (see Figure 3(B)) of wound closure at days 7, 10, and 12 post-surgery.

In relation to the wound closure, the wounds of the CG-Ag-Q sample group closed faster than those in CG-control group (see Figure 3(A)). Specifically, at day 7, the wound closure was approximately 54.4 ± 6.5% for the CG-control group and approximately 68.1 ± 13.2% for the CG-Ag-Q group. At day 10, the wounds had closed approximately 77.3 ± 8.6% and 85.4 ± 7.6% for the CG-control group and the CG-Ag-Q sample group, respectively. At day 12, the wounds in the CG-Ag-Q group nearly achieved complete closure (97 ± 2.6%) compared to those in the CG-control group (89.8 ± 2.3%).

**Figure 4:**
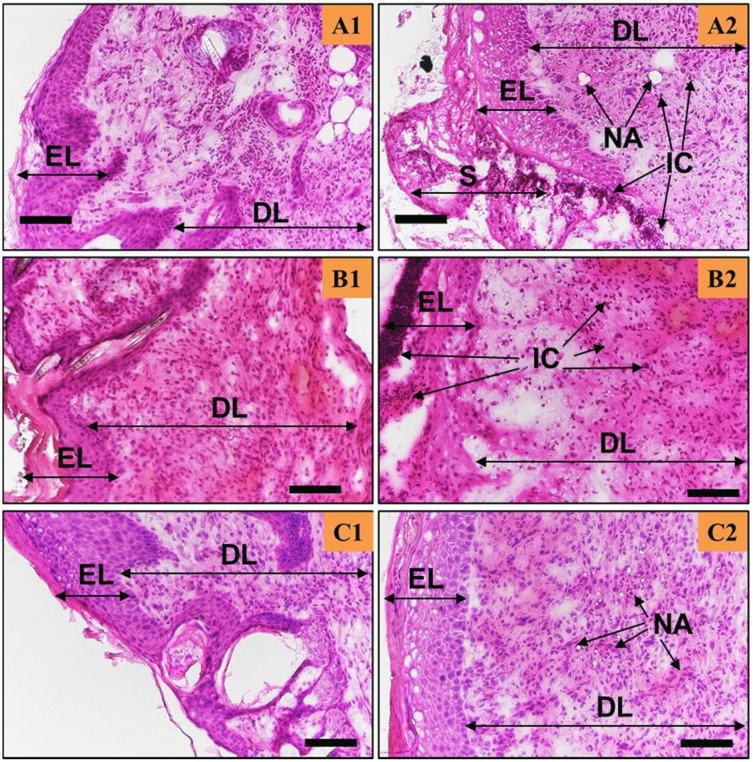
The H&E staining of the skin tissues at days 7, 10, and 12 post-surgery for the CG-control group. Scale bars are 100 µm. A, B, C show the staining sections at day 7, 10 12, respectively. (**A1**–**C1)** were from the areas near the wound edge and (**A2**–**C2)** were from the areas near the middle of the wounds. **Abbreviations:** EL, Epidermis; DL, dermis; S, scab; IC, inflammation-mediating cells; NA, neo-angiogenesis; SG, sebaceous glands.

**Figure 5:**
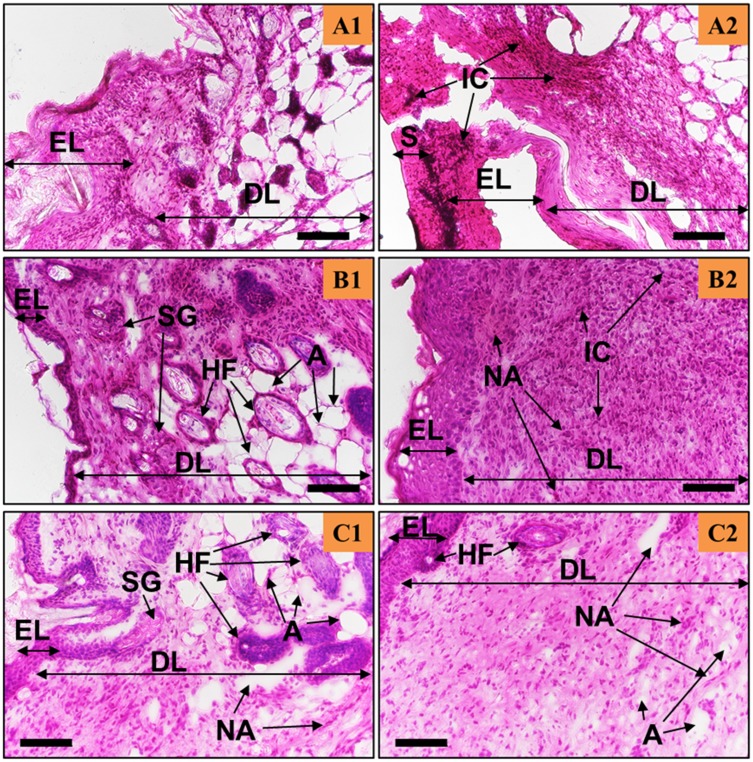
The Ag-Q coating improved tissue remodelling, neo-angiogenesis, and reduced inflammation. H&E staining of the skin tissues at days 7, 10, and 12 post-surgery for the CG-Ag-Q group. (**A1**–**C1)** were from the areas near the wound edge and (**A2**–**C2)** were from the areas near the middle of the wounds. Scale bars are 100 µm. **Abbreviations:** EL, Epidermis; DL, dermis; S, scab; IC, inflammation-mediating cells; NA, neo-angiogenesis; HF, hair follicles; SG, sebaceous glands; A, adipocytes.

The newly formed tissues were then studied using H&E staining (see Figures 4 and 5) to evaluate tissue remodelling, neo-angiogenesis, and inflammation. In general, the epidermis formed in both the CG-control (see Figure 4) and CG-Ag-Q (see Figure 5) groups, yet the wounds treated with CG-Ag-Q had presence of hair follicles (see Figure 5B and C) while the CG-control group did not.

In the CG-treated wounds, the inflammation-mediating cells, fibroblasts, and keratinocytes were still found at day 7 (see Figure 4A2 and B2) with a thick epidermis layer and the absence of the stratum basal layer. However, the epidermis was in the process of remodelling and blood vessels had formed (see Figure 4A2). At day 10, the number of inflammation-mediating cells decreased, and fibrotic tissues were clearly present (see Figure 4B). The thickness of epidermis reduced and hair was growing near the blood vessel (see Figure 4B1). Figure 4B1 also shows the formation of the stratum basal and that the epidermis was in the process of remodelling. Further, Figure 4B2 shows the formation of the sebaceous glands, which were close to the dermis layer. At day 12, the epidermis was thinner than at day 10 (see Figure 4B and C); the stratum basal of the epidermis was visible and there were numerous blood vessels (see Figure 4C2).

The wounds treated with the CG-Ag-Q dressing showed that the healing process occurred at a faster rate compared to that of wounds treated with CG (see Figures 4 and 5). Inflammation-mediating cells appeared to be fewer in the CG-Ag-Q than in the CG group (see Figure 5). Specifically, during the formation of the epidermis at day 7, the spinosum and granulosum had formed in the CG-Ag-Q group but were absent in the CG control group (see Figures 4A and 5A). Further, the epidermis had almost completed remodelling with the formation of the stratum basal layer at day 10 (see Figure 5B2). The external epithelium and hair follicles associated with glands formation were clearly observed (see Figure 5B1). At day 12, a thin dermis layer, as a normal tissue, was observed with the formation of glands and hair roots. The deposition of connective tissue and formation of adipose tissues was also enhanced (see Figure 5C2). In addition, the density of blood vessels was clearly higher in CG-Ag-Q-treated wounds than that in the CG-treated wounds (see Figure 5C). These results suggest CG-Ag-Q reduced the inflammation and enhanced the remodelling.

**Figure 6:**
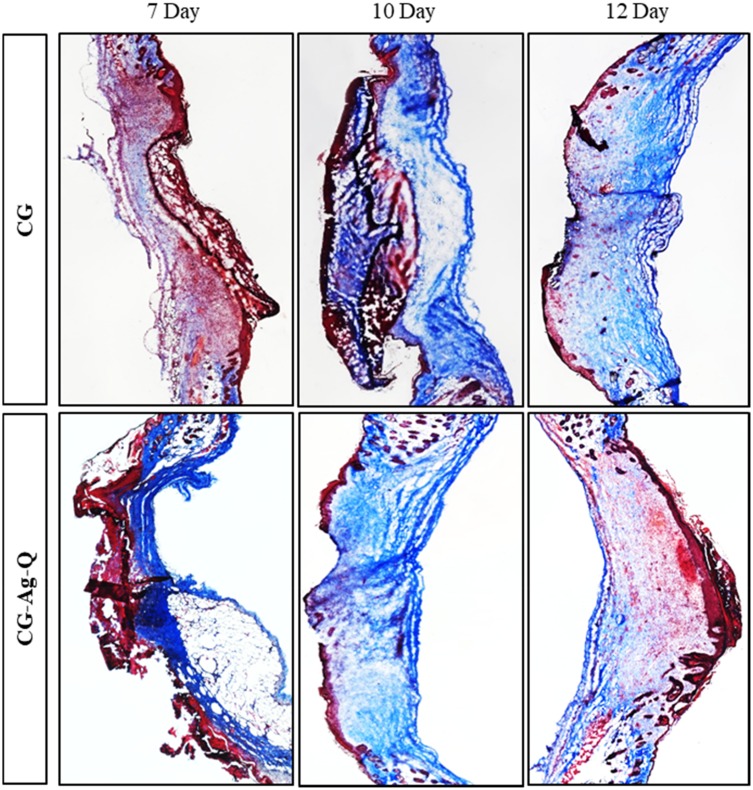
MT staining images of the skin tissues at days 7, 10, and 12 post-surgery in the CG and CG-Ag-Q groups.

MT staining was employed to evaluate the distribution of collagen matrix in the newly formed tissues (see Figure 6).

Figure 6 shows the collagen-rich regions of the CG-control and CG-Ag-Q-treated tissue sections at days 7, 10, and 12 post-surgery. In the CG-control group, collagen-rich regions were observed at day 7. These regions expanded significantly at days 10 and 12. In the CG-Ag-Q group, collagen appeared higher than in the CG control group for days 7 and 10, yet noticeably lower suggesting better remodelling at day 12.

## Discussion

This study developed a novel antibacterial and antioxidant coating for wound dressing materials that contained Ag and quercetin as the active ingredients. The coating was applied to a CG as a model dressing using a multistep dip-coating process. First, the Ag was immobilised onto the dressing via oxidation-driven polymerisation chemistry of PDA. Dopamine contains multifunctional groups of catechol and the amine rich proteins of marine mussels.^24^,^27^,^28^ It has been reported that DA possesses the ability to self-polymerise to form a nanometre thin coating on a wide range of substrates.^18^,^29^ When the CG was dipped in the DA solution in a mild alkaline and oxygen-rich environment, the DA polymerised to form a thin adherent layer of PDA on the CG surface. When immersed in silver nitrate solution, the incompletely oxidized active groups such as catechol and amine functional groups, presenting in the PDA layer reduced the silver ions into a surface-bound metallic Ag.^8^,^18^ Two hydroxyl groups in the ortho positions on the PDA layer were likely involved in a reaction whereby the dihydroxyl molecules lost two-electrons to form a quinone structure and stabilise the newly formed Ag.^30^,^31^

To incorporate quercetin into the CG-Ag dressing, a commercially available medical grade methoxyl-amine-functionalised silicone was used as a binding agent. This agent was selected because its lubricating property would also reduce the tendency of the dressing to adhere too strongly to the wound surface. The silicone coating also regulated the release of bound quercetin and Ag, providing extended activity for up to 8 days (see Figure 1(C).

A gram-positive (*S. aureus*) pathogen was used in this study. It was selected because it is a major pathogen in infected wounds.^32^–^34^ *S. aureus* is also one of the most difficult pathogens to treat and is a common type of human wound bacteria that can be isolated from soft tissue injuries regardless of the initial causes. The in vitro antibacterial assay results showed that the synergism of Ag and quercetin enhanced the antibacterial ability of quercetin.

Wound healing comprises four phases including haemostasis, inflammation, proliferation, and remodelling.^35^ The ROS of post-cutaneous wounds may have a supportive effect on wound repair as well as signal transduction in the re-epithelialisation and proliferation of cells at a low or normal level. Despite that, high levels of ROS might damage intercellular macromolecules caused by the oxidative stress.^36^ Quercetin is known as a scavenger of ROS and reactive nitrogen species that include superoxide anion radical, nitric oxide and peroxynitrite anion.^37^ This property was therefore strongly linked with CG-Q-Ag group’s enhanced wound closure and tissue remodelling.

In our study, wound closure was primarily due to wound contraction in which cells migrate from the wound edge.^38^ It has been shown by other research groups that quercetin could enhance myofibroblast activity and increase epithelial cell growth.^39^ Thus, the faster wound closure in the CG-Ag-Q group could be attributed to the increased activity of myofibroblasts during wound healing process.^40^

Compared to the CG-control group, the CG-Ag-Q group showed a decrease in inflammation-mediating cells and significantly higher collagen deposition at the early stages of healing suggesting increased fibroblast functions. The results also revealed that the CG-Ag-Q treatment might induce the migration and proliferation of keratinocytes as evident through the mature epithelium layer.^41^

## Conclusion

In this study, a new antimicrobial-antioxidant coating was developed and applied to dressing materials that were used to aid the healing of wounds. The active ingredients of the coating were Ag and quercetin which were applied to a cotton gauge as a model dressing material using a dip-coating process. In vitro assays showed the antimicrobial of quercetin against *S. aureus* was significantly enhanced when used in combination with Ag. Strong antioxidant activity was also demonstrated. The dressing was applied to surgical wounds created on mice and showed faster wound closure, reduced inflammation, enhanced neo-angiogenesis, increased collagen deposition and remodelling. This new coating thus should be further investigated as a promising material for wound dressing applications.
